# Compound heterozygous and digenic inheritance in early-onset Parkinson’s disease: a systematic review

**DOI:** 10.3389/fnagi.2026.1930518

**Published:** 2026-10-22

**Authors:** Victor Flores-Ocampo, Grecia Sevilla-Parra, Fangyuan Cao, Deepak Darshan, Santiago Díaz-Torres, Claudia Gonzaga-Jauregui, Kishore Raj Kumar, Miguel Enrique Rentería, Natalia S. Ogonowski

**Affiliations:** 1 Brain & Mental Health Program, QIMR Berghofer Medical Research Institute, Brisbane, QLD, Australia; 2 School of Biomedical Sciences, Faculty of Health, Medicine and Behavioural Sciences, The University of Queensland, Brisbane, QLD, Australia; 3 Laboratorio Internacional de Investigación sobre el Genoma Humano, Universidad Nacional Autónoma de México, Querétaro, Mexico; 4 Population Health Program, QIMR Berghofer Medical Research Institute, Brisbane, QLD, Australia; 5 Department of Neurology and Molecular Medicine Laboratory, Concord Repatriation General Hospital, Concord, NSW, Australia; 6 ANZAC Research Institute, University of Sydney, Sydney, NSW, Australia; 7 Garvan Institute of Medical Research, Darlinghurst, NSW, Australia; 8 School of Clinical Medicine, UNSW Medicine & Health, St Vincent's Healthcare Clinical Campus, Faculty of Medicine and Health, UNSW, Sydney, NSW, Australia; 9 School of Biomedical Sciences, Faculty of Health, Queensland University of Technology, Brisbane, QLD, Australia

**Keywords:** compound heterozygosity, digenic inheritance, early onset Parkinson’s disease (EOPD), genetics of Parkinson’s disease, Parkinson’s disease

## Abstract

**Introduction:**

Compound heterozygous (CH) genotypes represent an established biallelic autosomal-recessive mechanism in early-onset Parkinson’s disease (EOPD), whereas digenic inheritance (DI) has been proposed for variant combinations across two genes but is more difficult to establish. We systematically evaluated the genetic architecture and clinical features of CH cases and cases reported as DI in the published literature.

**Methods:**

This PRISMA-compliant systematic review included studies reporting CH or DI EOPD through September 2026. Two reviewers independently screened studies, extracted data, and curated variant- and case-level information. Variants were harmonized using current Human Genome Variation Society nomenclature and classified by gene, variant type, ancestry, and inheritance model.

**Results:**

We included 106 studies comprising 304 CH and 16 DI cases. CH was dominated by *PRKN*, with structural variants present in 85% (205/241) of *PRKN* cases, predominantly exon-level deletions. *PINK1* was the second most frequently implicated CH gene, whereas other CH subtypes were rare and genetically heterogeneous. Cases reported as DI involved seven genes, most commonly *LRRK2*–*PRKN* and *PINK1*–*PRKN*, although most variant combinations were unique. Across the CH and reported-DI groups, patients presented with a core Parkinsonian syndrome but showed substantial variability in age at onset, motor severity, and non-motor manifestations. *PRKN*-associated CH cases generally had earlier onset and favorable levodopa responsiveness, whereas *PLA2G6*- and *VPS13C*-associated cases more often exhibited cognitive impairment, psychiatric features, or structural neuroimaging abnormalities. No reproducible genotype-specific phenotype emerged.

**Conclusion:**

Compound heterozygous represents a conventional biallelic recessive mechanism in EOPD and was largely driven by *PRKN* structural variation. In contrast, cases reported as DI were rare and heterogeneous, and the available evidence generally did not establish causal digenic inheritance. These findings support comprehensive detection of structural and sequence variants while highlighting the need for segregation, functional, and replication evidence to distinguish causal DI from modifier effects or coincidental variant co-occurrence.

## 1 Introduction

Early-onset Parkinson’s disease (EOPD), defined by symptom onset before 50 years of age (Mehanna et al., 2025), is enriched for monogenic forms and provides a key framework for investigating the genetic architecture of Parkinson’s disease (PD) (Lesage and Brice, 2009; Hickman et al., 2025). Although core clinical features overlap with late-onset Parkinson’s disease (LOPD), younger individuals often exhibit a distinct disease course and face specific therapeutic challenges. Monogenic PD is broadly classified into autosomal dominant and autosomal recessive forms. Dominant variants, particularly in *LRRK2* and *SNCA*, are more commonly associated with LOPD, whereas recessive loss-of-function variants in *PRKN, PINK1, PARK7*, and *VPS13C* constitute the most frequent genetic causes of EOPD (Mehanna et al., 2014; Jia et al., 2022).

Autosomal recessive PD is typically attributed to biallelic pathogenic variants, either homozygous or compound heterozygous (CH), within a single gene, with CH referring to two different pathogenic variants occurring in *trans* on opposite alleles (Guadagnolo et al., 2021). CH is a recognized mechanism and is often associated with earlier onset and variable clinical expression in familial EOPD cohorts. However, this model does not account for all cases. The co-occurrence of heterozygous variants across multiple PD-related genes suggests that digenic or broader oligogenic mechanisms, including modifier effects, may contribute to disease susceptibility and expression. Digenic inheritance (DI) refers to pathogenic variants in two distinct genes whose combined effects contribute to the clinical phenotype. In EOPD, such cases are typically inferred and may reflect either biological interaction between genes or the coexistence of a primary pathogenic variant with a modifier allele (Guadagnolo et al., 2021).

Resources such as the Movement Disorder Society Gene (MDSGene) (Lill et al., 2016) database systematically catalog genotype–phenotype associations and annotate CH variants within gene-specific entries. However, their focus on established EOPD genes and diagnosis-driven classifications may limit the identification of CH variants beyond canonical loci and restrict cross-gene comparisons. In contrast, DI remains rare and poorly characterized. Although interactions between *PINK1* and *PRKN* have been explored (Han et al., 2023), data on other gene combinations and their frequency are limited. In addition, the absence of consistent definitions and reporting standards further complicates the distinction between true DI and coincidental variant co-occurrence. Together, these limitations hinder systematic evaluation of combinatorial genetic models in EOPD.

To address this gap, we systematically examined CH and reported DI cases in EOPD across the published literature. We explored variant-level patterns, gene-pair frequencies, ancestry-specific distributions, and genotype–phenotype associations. Because no unified synthesis of CH and DI was available, and robust estimates of their frequency in large, unbiased cohorts were lacking, we curated and harmonized reported cases, standardized variant annotations, and performed systematic comparisons across genes and populations. Our aim was to characterize the genetic and clinical features of each category in EOPD.

## 2 Methods

### 2.1 Systematic review

A systematic review was performed in accordance with the Preferred Reporting Items for Systematic Review and Meta-analyses (PRISMA) guidelines (Page et al., 2021) to identify reported cases of EOPD classified as compound heterozygous (CH) or digenic inheritance (DI). PubMed was searched on 8 September 2026 using two complementary search strategies targeting CH and DI. The strategies incorporated broader terminology, biallelic heterozygous, in *trans*, oligogenic, multilocus, and modifier variant/allele terms. The searches covered publications from 1 January 1900 to 8 September 2026, with no additional language, article type, or geographic restriction. Records were combined and deduplicated before screening. Titles and abstracts were screened first, allowed by full-text assessment by two independent reviewers, with disagreements resolved by consensus. The complete search strategies, eligibility criteria, study-selection procedures, and PRISMA 2020 checklist are available in the Supplementary Method S1 and Figure 1.

**Figure 1:**
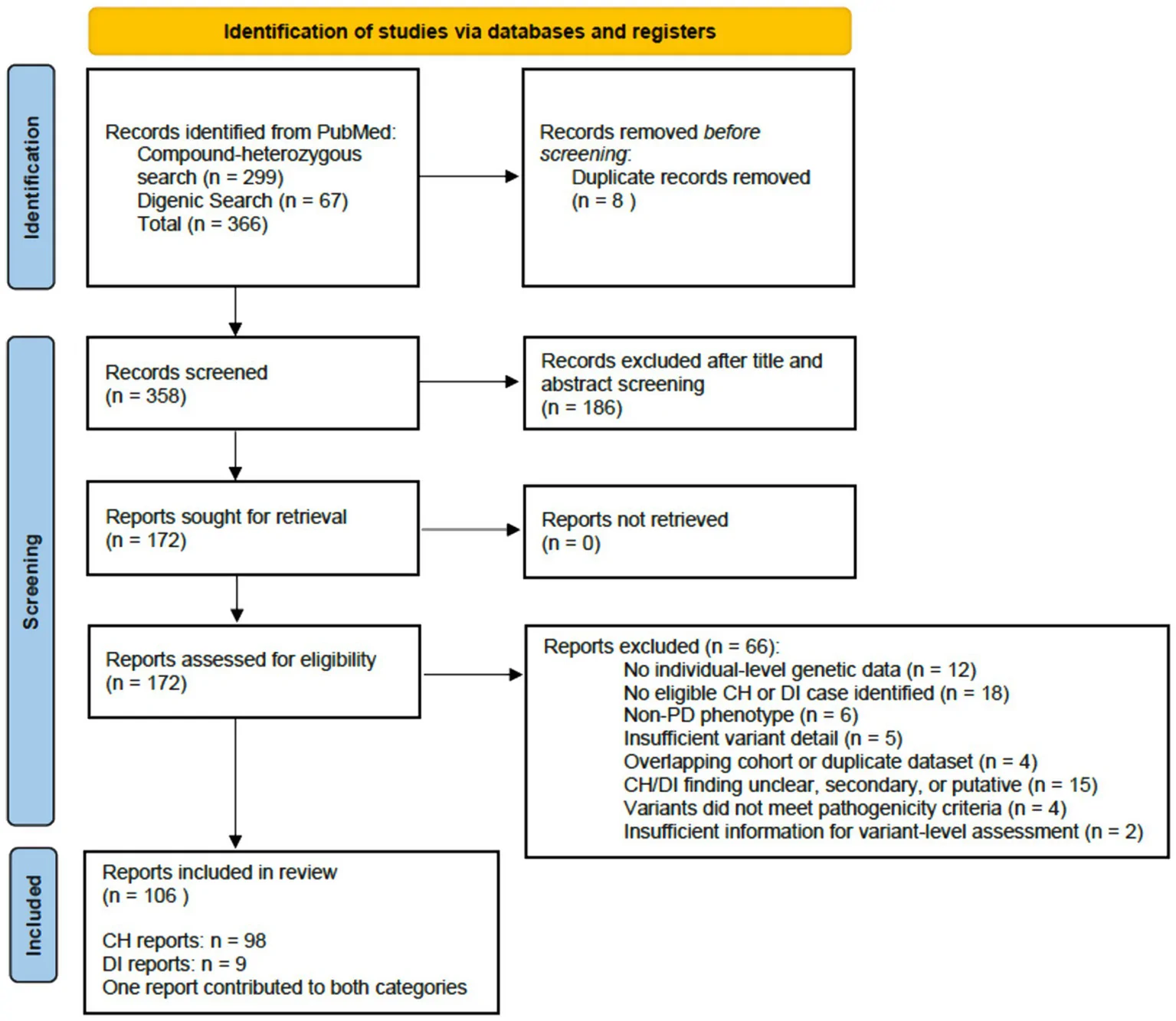
PRISMA flow diagram of study selection: flow diagram of study identification, screening, eligibility, and inclusion for compound heterozygous (CH) and digenic inheritance (DI) cases in early-onset Parkinson’s disease (EOPD). The PRISMA flow diagram was adapted from the PRISMA 2020 template to present our study selection process (Page et al., 2021). Creative Commons Attribution 4.0 International (CC BY 4.0). Source: https://www.prisma-statement.org/prisma-2020-flow-diagram.

#### 2.1.1 Inclusion and exclusion criteria

Eligibility criteria were defined *a priori*. Studies were considered eligible if they reported individuals with a primary diagnosis of EOPD classified as having CH or DI, with sufficient individual-level genetic information to identify the relevant variants and assign them to the reported case. Eligibility was based on the classification of cases as EOPD or young-onset PD in the source publication. Age at onset was extracted where available but was not applied retrospectively as an additional eligibility threshold. CH was defined as two distinct variants within the same gene reported as constituting a CH genotype. DI was defined as variants in two different genes reported as jointly contributing to the phenotype or as constituting a digenic genotype. Classification was based on the inheritance model reported in the original publication. We did not independently adjudicate the causal contribution of individual variant combinations, perform *de novo* ACMG/AMP classification, or reassess the molecular diagnosis of included cases.

Exclusion criteria comprised studies describing CH or DI cases as purely “putative”; duplicate reports of previously included cases; studies focusing on parkinsonism or alternative phenotypes without a primary diagnosis of PD; studies that did not clearly distinguish between CH and DI carriers or lacked sufficient genetic detail to determine zygosity or genotype; and reports in which the relevant variant combinations included one or more variants classified as benign or likely benign. Variants of uncertain significance (VUS) were retained when supported by additional evidence, whereas a VUS, conflicting ClinVar classification, or absence of a ClinVar entry did not, in isolation, warrant exclusion. No case was excluded solely because structural-variant phase had not been experimentally resolved, provided that the source publication reported the variants as constituting a CH genotype and supplied sufficient variant-level information.

Duplicate records were removed prior to screening. Titles and abstracts were reviewed to exclude studies that did not meet the inclusion criteria, followed by full-text assessment for eligibility. From all included studies, data were systematically extracted using a standardized framework derived from the data extraction table.

#### 2.1.2 Data extraction

Relevant data from included articles were extracted by a pair of independent authors (V. F-O, N. S. O) into a standardized extraction template. Extracted variables included genetic information (genes involved, variant type and classification, and inheritance model), demographic (age at onset, sex, and ancestry), and detailed phenotype features. Clinical data were extracted and organized into motor and non-motor domains. Motor features included core Parkinsonian symptoms (rigidity, bradykinesia, tremor, postural instability, and dystonia); additional motor manifestations; and treatment-related complications, as well as standardized measures of disease severity (Movement Disorder Society–Unified Parkinson’s Disease Rating Scale [MDS-UPDRS], UPDRS III, and Hoehn and Yahr stage). Non-motor features were grouped into autonomic, psychiatric, cognitive, and sleep-related domains. Given variability in reporting across studies, individual symptoms were extracted when available and categorized accordingly. Additional clinical data included pharmacological and advanced therapeutic interventions, neuropsychological assessments, and ancillary investigations. Neuroimaging findings (structural and functional), electrophysiological studies, laboratory tests, and neuropathological data were also collected when reported. For clinical variables, features were recorded as present only when explicitly reported in the source publication; lack of reporting was not interpreted as absence of the feature. Because clinical reporting was incomplete and non-standardized, phenotype comparisons across gene groups were descriptive; no inferential tests were performed. A summary of characteristics of included studies can be found in Tables 1–3.

**Table 1:** Age at onset of EOPD CH cases stratified by sex and continental ancestry.

Participant group	Total, eligible *N*	AAO available *N*	AAO missing *n*	AOO, mean (SD)	AOO, median	AOO, min-max	Top three predominant genes (number of studies reporting gene)
Overall	304 (100%)	261	43	31.22 ± 11.46	31	6–76	*PRKN (66 studies), PINK1 (12 studies), FBXO7 (5 studies)*
Males	110 (36.2%)	104	6	32.87 ± 11.8	31,5	6–72	*PRKN (37 studies), FBXO7 (5 studies), PINK1 (4 studies)*
Females	111 (36.5%)	108	3	28.04 ± 10.5	27	8–67	*PRKN (43 studies), PINK1 (7 studies), VPS13C (3 studies)*
Sex Not reported	83 (27.3%)	49	34	36.88 ± 10.55	38	9–76	PRKN (9 studies), PINK1 (2 studies), VPS13C (2 studies)
Ancestry distribution							
AFR	5 (1.6%)	4	1	34.5 ± 19.12	33	16–56	*PRKN (2 studies), SYNJ1 (1 study)*
AMR	30 (9.9%)	30	0	36.63 ± 7.28	39	15–44	*PRKN (5 studies), PINK1 (1 study)*
EAS	87 (28.6%)	72	15	25.83 ± 9.66	25	6–54	*PRKN (22 studies), PINK1 (4 studies), PLA2G6 (4 studies)*
EUR	173 (56.9%)	146	27	32.47 ± 11.94	33	8–76	*PRKN (35 studies), PINK1 (7 studies), VPS13C (4 studies)*
MDE	8 (2.6%)	8	0	36 ± 10.56	34	21–50	*PRKN (2 studies), FBXO7 (1 study)*
NA	1 (0.3%)	1	0	24	24	24–24	PRKN (1 study)

AOO, age of onset; SD, standard deviation; AFR, African; AMR, American; EAS, East Asian; EUR, European; MDE, Middle Eastern.

**Table 2:** Clinical phenotype, motor severity, and neuropsychological assessment in CH EOPD.

Motor features			
Feature	^&^Cases reported as present, n	Genes	References
Bradykinesia	67	*PRKN, VPS13C, PLA2G6, FBXO7, PINK1, DJ-1, EPG5, SYNJ1, POLG*	19, 24, 25, 27–30, 37–40, 42, 46, 47, 50–52, 55, 57, 60, 61, 65, 67, 76, 81, 82, 93–95, 99, 101, 102, 108, 113, 116, 117
Akinesia	10	*VPS13C, PRKN, PINK1*	30, 41, 50, 54, 59
Rigidity	68	*PRKN, PINK1, PLA2G6, VPS13C, FBXO7, DJ-1, EPG5, SYNJ1, POLG*	19, 24, 27, 28, 30, 35, 39, 42, 46, 47, 50–52, 54, 55, 57, 60, 61, 65, 71, 75, 76, 80–82, 86, 89, 90, 93, 95, 99, 102, 107, 108, 115, 117
Tremor	59	*PRKN, PINK1, FBXO7, DNAJC6, PLA2G6, SYNJ1, POLG*	19, 24, 25, 27, 35, 37, 40, 41, 47, 49–51, 54, 55, 61, 67, 71, 75, 78–81, 86, 90, 93–95, 99, 101, 108, 113, 116
Resting tremor	38	*PRKN, PINK1, VPS13C, FBXO7, PLA2G6, EPG5, SYNJ1, POLG*	19, 27, 29, 37, 39, 40, 42, 46, 50, 52, 54, 57, 61, 65, 75, 76, 81, 90, 93, 95, 99, 107, 108
Essential Tremor	1	*PRKN*	40
Postural instability	33	*PRKN, PLA2G6, DJ-1, EPG5, SYNJ1, POLG*	24, 25, 28, 38, 40, 42, 50, 54, 55, 57, 61, 71, 78–81, 86, 99, 108, 117
Dystonia	27	*VPS13C, PRKN, FBXO7, PLA2G6, PINK1, SYNJ1, POLG*	19, 24, 30, 38, 40–42, 50–52, 55, 59, 61, 65, 71, 76, 79, 81, 95, 99, 102
Dyskinesia	27	*PRKN, VPS13C, DNAJC6, EPG5, POLG*	35, 38, 50, 51, 55, 57, 67, 78, 79, 89, 90, 93, 120
Gait disturbance	34	*VPS13C, PRKN, PLA2G6, FBXO7, PINK1, SYNJ1, POLG*	19, 25, 28, 29, 35, 38, 40, 41, 50, 55, 58, 59, 65, 79–81, 85, 86, 95, 99, 102, 108, 115, 116
Slow movement	16	*PRKN, PINK1, FBXO7, PLA2G6, SYNJ1*	24, 45, 50, 52, 59–61, 75, 81, 95, 99, 107, 113
Hypomimia	16	*PRKN, PLA2G6, VPS13C, PINK1, DJ-1, SYNJ1, POLG*	30, 37, 39, 41, 59, 60, 65, 81, 90, 107, 108, 113, 116, 117
Hypophonia	5	*DJ-1, PRKN, SYNJ1, POLG*	55, 59, 81, 90, 117
Freezing of gait	4	*PRKN*	50, 76
Wearing off (motor complications of dopaminergic therapy)	11	*VPS13C, PRKN, DNAJC6, PINK1*	30, 38, 40–42, 50, 61, 65, 79, 89

Non-motor features				
Category	Clinical feature	^&^Cases reported as present, *n*	Genes	References
Autonomic	Constipation	12	*PRKN, EPG5, FBXO7*	19, 24, 35, 57, 107, 108, 113
	Olfactory impairment	6	*PRKN, EPG5*	57, 107
Psychiatric	Depression	19	*PRKN, VPS13C, PINK1, FBXO7*	19, 24, 29, 30, 35, 41, 46, 59, 65, 78, 79, 107, 113, 115, 120
	Anxiety	9	*VPS13C, PRKN, PINK1*	19, 29, 35, 39, 50, 59, 79, 107
	Obsessive compulsive disorder	1	*PRKN*	35
	Psychosis (hallucinations/delusions)	9	*VPS13C, PLA2G6, EPG5, PRKN, POLG*	29, 30, 52, 57, 78, 90
	Impulsive-control disorder	3	*PRKN, FBXO7*	40, 78, 113
Cognitive	Mild cognitive impairment	12	*PRKN, DJ-1, EPG5, POLG*	25, 35, 57, 78, 90, 107, 117
	Dementia	6	*PRKN, PLA2G6, EPG5*	52, 57, 79
	Memory impairment	2	*VPS13C*	29, 30
	Handwriting impairment	6	*FBXO7, PRKN, VPS13C*	25, 30, 59, 60, 95
Speech	Speech impairment	3	*VPS13C, SYNJ1*	29, 81
	Dysarthria	3	*VPS13C, PLA2G6*	29, 52
Sleep	Sleep disturbance	14	*PRKN, PINK1, PLA2G6, EPG5, FBXO7*	35, 57, 59, 65, 78, 108, 113, 115, 116, 120
	Restless legs syndrome	3	*PRKN, PINK1*	19, 39, 50
	Fatigue	3	*PLA2G6, PRKN*	28, 99
Neurological signs	Hyperreflexia	4	*PRKN*	25, 108, 115
	Weakness	3	*PRKN*	59, 80
	Ataxia	2	*PLA2G6*	52
Sensory	Pain	5	*PRKN*	42, 99, 115

Motor severity				
Scale	Score range (min–max)	^&^Cases reported as present, n	*Genes	References
MDS-UPDRS	11–92	31	*PRKN, PINK1, FBXO7, EPG5, SYNJ1, POLG*	24, 39, 41, 42, 46, 55, 57, 59, 61, 75, 81, 93, 95, 99, 101, 108
UPDRS I	0–19	9	*PLA2G6, PRKN*	28, 49, 86, 120
UPDRS II	0–38	11	*PLA2G6, PRKN*	28, 49, 76, 86, 120
UPDRS III	0–62	31	*PRKN, PLA2G6, GBA1, PINK1, DJ-1*	26, 28, 40, 50, 63, 65, 67, 76, 79, 86, 107, 116, 117, 120
UPDRS IV	0–11	9	*PLA2G6, PRKN*	28, 49, 86, 120
Hoehn and Yahr stage	1–5	39	*PRKN, PLA2G6, GBA1, EPG5, SYNJ1*	24, 28, 38, 42, 46, 57, 61, 63, 76, 81, 93, 94, 99, 108, 116, 120

Cognitive and neuropsychological scales				
Scale	Values reported	^&^Cases reported as present, n	*Genes	References
MMSE				
Normal	24–30	12	*PRKN, VPS13C, PINK1, DJ-1, EPG5, SYNJ1, FBXO7*	24, 30, 42, 47, 57, 61, 65, 76, 81, 93, 94, 101, 107, 113, 116, 117
Mild impairment	18–23	8		
Moderate–severe impairment	<18	4		
MoCA				
Normal	≥26	5	*PRKN, PLA2G6, FBXO7, EPG5*	24, 28, 42, 93, 95, 107, 115, 116
Impaired	<26	9		
HAMD				
Normal	0–7	3	*PRKN, PLA2G6*	24, 28, 42, 46, 61, 116
Mild	8–16	1		
Moderate	17–23	2		
Severe	≥24	1		
HAMA				
Mild	<17	2	*PRKN, PLA2G6*	24, 28, 61, 116
Mild–moderate	18–24	1		
Moderate–severe	25–30	1		

*Genes reported correspond to cases in which the respective clinical features or scores were available.

^&^Counts represent the number of individuals in whom the finding was explicitly reported. Because explicit absence and assessment status were inconsistently reported, these counts should not be interpreted as prevalence estimates.

Reference numbers correspond to the ‘Ref index’ column in Supplementary Tables 1, 4, which provide the corresponding DOI and PMID.

**Table 3:** Therapeutic approaches and diagnostic findings in CH EOPD.

Therapeutic approaches				
Treatment category	Treatment	^&^Cases reported as present, *n*	Genes	References
Dopaminergic therapies	Levodopa (including combinations*)	83	*PRKN, PINK1, PLA2G6, VPS13C, DJ-1, FBXO7, POLG, SYNJ1*	24, 25, 27, 28, 30, 37, 38, 40, 42, 46, 47, 50–52, 54, 55, 57–61, 65, 75, 76, 78–82, 93, 101, 102, 108, 113, 117
	Dopamine agonists	6	*PRKN, POLG*	47, 54, 79, 90
	Pramipexole	10	*PRKN, PINK1, FBXO7, POLG*	24, 40, 55, 78, 90, 108, 113, 117
	Ropinirole	2	*PRKN*	38, 79
	Rotigotine	1	*PINK1*	41
	Cabergoline	1	*PRKN*	50
	Piribedil	1	*PLA2G6*	28
Neuropsychiatric and Cognitive treatments	Sertraline	1	*PINK1*	75
	Rivastigmine	1	*VPS13C*	30
	Memantine	1	*VPS13C*	30
Advanced and supportative therapies	Deep brain stimulation	5	*VPS13C, PRKN, DNAJC6*	38, 40, 59, 89
	Dopaminergic therapy	4	*DNAJC6, PRKN*	51, 82
	PEG (severe dysphagia)	3	*VPS13C*	30
	pyridoxine	1	*PRKN*	51
Adjunct and symptomatic therapies	Amantadine	3	*PLA2G6, PRKN, FBXO7*	28, 79
	Propranolol	1	*PRKN*	40
	Botulinum toxin injection	1	*PLA2G6*	52
	MAO-B inhibitors^§^	6	*PRKN, PINK1*	40, 79, 101, 113
	Anticholinergics^‡^	5	*PLA2G6, PRKN*	28, 37, 51, 93

Neuroimaging, neuropathological and ancillary findings				
Treatment category	Finding	Interpretation	Genes	References
Structural MRI	Normal MRI	No structural abnormalities	*PRKN, PINK1, PLA2G6, DJ-1, SYNJ1, POLG*	25, 27, 28, 38, 40, 61, 65, 67, 75, 81, 90, 107, 117
	Cortical and cerebellar atrophy	Predominantly frontal cortical atrophy with temporal and parietal involvement; mild–moderate cerebellar atrophy.	*VPS13C, PLA2G6, PRKN, EPG5, POLG*	28–30, 37, 42, 52, 55, 57, 93, 99, 116
	Basal ganglia abnormalities	Hypointensity in globus pallidus and putamen, consistent with iron deposition	*VPS13C, PLA2G6, PRKN, EPG5, POLG*	28–30, 37, 42, 52, 55, 57, 93, 99, 116
	Advanced MRI markers (3T/7T)	Loss of nigral swallow tail sign, reduced neuromelanin signal, increased iron deposition	*PINK1*	39
	Substantia nigra changes	Signal alterations and atrophy suggestive of nigral degeneration	*VPS13C, PLA2G6, PRKN, EPG5, POLG*	28–30, 37, 42, 52, 55, 57, 93, 99, 116
	Cerebellar atrophy (advanced imaging – MP2RAGE)	Reduced cerebellar volume and white matter tract density	PLA2G6	28
Metabolic imaging (FDG-PET)	Cortical hypometabolism	Involvement of frontal, temporal, parietal, occipital cortex, thalamus, putamen, and cerebellum	*VPS13C, PRKN, PLA2G6, PINK1*	29, 30, 41, 42, 52, 58
	Mixed metabolic pattern	Cortical hypometabolism with relative hypermetabolism in precuneus/posterior cingulate and ventral striatum	PRKN, PLA2G6, PINK1	41, 42, 52, 58
Dopaminergic imaging	DAT-SPECT (including DaTSCAN/FP-CIT)	Reduced striatal dopamine transporter uptake, often asymmetric (putamen > caudate, right-predominant)	*PRKN, PINK1, VPS13C, POLG*	30, 38–41, 90, 115
	TRODAT/DAT PET/18F-DOPA	Reduced dopaminergic uptake in putamen and caudate nucleus.	*PRKN, EPG5*	27, 42, 46, 57, 58
	IMP-SPECT	Reduced basal ganglia signal	*PRKN*	38
	SPECT (non-specific)	Normal or non-diagnostic findings	*PRKN*	38
Neuropathology	α-synuclein pathology	Lewy bodies and neurites in neocortex, limbic regions, brainstem	*VPS13C*	29
	Tau pathology	Variable, ranging from limited hippocampal involvement to advanced (Braak stage VI)	*VPS13C*	29
	β-amyloid deposition	Extensive β-amyloid plaque deposition.	*VPS13C*	29
	Post-mortem brain findings	Cortical atrophy and severe nigral neuronal loss	*VPS13C*	29
Neurophysiology	EMG	Predominantly sensory axonal neuropathy.	*PRKN, POLG*	55, 59, 90
	EEG	Non-specific abnormalities	*PLA2G6*	116
Others	Copper/ceruloplasmin	Normal.	*PRKN, PLA2G6, POLG*	55, 107, 116
	Skin biopsy	no alpha-synuclein pathology	*DJ-1*	117
	CSF	Normal routine parameters (no inflammatory abnormalities)	*PRKN*	115
	CT	Normal	PRKN	27
	PET/CT	Reduced striatal signaling with additional ischemic lesions (frontal/parietal)	PRKN	27, 42

^*^Includes levodopa combined with carbidopa, benserazide, co-careldopa, and Medopar.

^§^Includes rasagiline and selegiline.

^‡^Includes benzhexol, trihexyphenidyl, and biperiden.

^&^Counts represent the number of individuals in whom the finding was explicitly reported. Because explicit absence and assessment status were inconsistently reported, these counts should not be interpreted as prevalence estimates.

Reference numbers correspond to the ‘Ref index’ column in Supplementary Tables 1, 4, which provide the corresponding DOI and PMID.

### 2.2 Genetic data curation and variant harmonization

Genetic data were curated for each reported CH and DI case following study selection. To account for related individuals, each case was assigned both a family identifier (FID) and a unique individual identifier (IID) (Supplementary Tables 1, 4).

All reported variants were extracted in their original notation and mapped to the corresponding genes. To address heterogeneity in variant reporting across studies, variants were standardized according to Human Genome Variation Society (HGVS) nomenclature (den Dunnen et al., 2016) using RefSeq transcripts. Curation was performed at the individual-variant level, incorporating cross-referencing with previously published reports, identification of transcript references used in the source studies, and alignment of legacy nomenclature to current HGVS-compliant formats. Where available, variants were linked to dbSNP identifiers.

Structural variants were harmonized at the exon level (e.g., deletions, duplications, inversions), reflecting the limited resolution reported in the primary studies. Variants were further aligned with ClinVar records (Landrum et al., 2020) when available, and corresponding CPRA (Chromosome, Position, Reference, Alternate) identifiers were retrieved using SPDI (Sequence, Position, Deletion, Insertion) notation (Holmes et al., 2020) or obtained through Franklin (Genoox) (Introduction to Franklin, 2025) for variants not represented in ClinVar.

Following harmonization, single-nucleotide variants (SNVs) and small insertions and deletions were represented on the GRCh38/hg38 human-genome reference assembly and annotated with genomic coordinates, reference and alternate alleles, and HGVS-compliant designations using RefSeq transcripts where these could be established. Indels were left-aligned and normalized against the GRCh38 reference sequence using bcftools v1.20 (Li, 2011), with subsequent functional annotation performed using ANNOVAR (Wang et al., 2010) with the hg38 build, RefGene, ClinVar (21 July 2025 release), and dbNSFP v4.7a (Liu et al., 2020). Variants reported using different nomenclatures but representing identical molecular events were consolidated under unified identifiers.

As part of quality control, source-reported and harmonized variant designations were assessed for consistency across the assigned gene, transcript, genomic coordinates, reference and alternate alleles, and predicted molecular consequence. Automated checks were used to identify incompatible frameshift/in-frame or stop-gain annotations, transcript or gene mismatches, coordinate or allele inconsistencies, and deletion/duplication discrepancies. Flagged entries were manually reviewed against the source publication and available transcript- and genomic-level information. Variants for which an unambiguous molecular representation could not be established were excluded from quantitative variant-level analyses.

The complete harmonization and audit procedures, including the original-to-harmonized mappings and corresponding QC outcomes, are provided in the Supplementary Methods and Supplementary Tables. Downstream frequency analyses of individual variants and variant pairs were conducted in R v4.4.0 using custom scripts.

## 3 Results

### 3.1 Study selection

The initial PubMed search identified 366 records, 299 for CH and 67 for DI. After PRISMA screening, a total of 106 unique articles were included (98 for CH and 9 for DI, with one study contributing to both categories). The selection process is depicted in the flowchart in Figure 1. CH studies were published between 2001 and 2025 and predominantly comprised case reports and original articles. A total of 80 CH studies had previously been curated in the MDSGene database under the “*early-onset Parkinson’s disease, recessive forms*” category, whereas 18 studies had not yet been included. Across both CH and DI studies, genetic reporting was heterogeneous, particularly with respect to variant nomenclature and transcript usage (Supplementary Tables 1–4).

Clinical and demographic data were inconsistently reported across CH studies. Age at onset was available for 261 out of 304 individuals (86%) and ranged from 6 to 76 years, with a mean age at onset of 31.22 years. 13/261 individuals (5.0%) with available data had onset at or after 50 years. Sex was reported for 221 out of 304 (73%) of cases, and 110 (50%) individuals with available sex data were male. Continental ancestry was reported for 303/304 individuals (99.7%; one missing). Of the total cohort, 173/304 (56.9%) were of European ancestry (EUR), followed by 87/304 (28.6%) East Asian (EAS), 30/304 (9.9%) admixed American (AMR), 8/304 (2.6%) Middle Eastern (MDE), and 5/304 (1.6%) African (AFR) (Table 1). Among individuals with available age-at-onset data, mean onset was earliest in EAS cases (72/87 available; 15 missing; mean 25.83 years, SD 9.66) and latest in AMR (30/30 available; none missing; mean 36.63 years, SD 7.28) and MDE cases (8/8 available; none missing; mean 36.00 years, SD 10.56). Corresponding estimates were 32.47 years (SD 11.94) among EUR cases (146/173 available; 27 missing) and 34.50 years (SD 19.12) among AFR cases (4/5 available; one missing). Across all ancestry groups, *PRKN* was the most frequently reported gene, whereas *PLA2G6* and *PINK1* were more common in EAS populations and *FBXO7* predominated in MDE cases.

Nine DI studies, published between 2006 and 2023, were included. Reporting of genetic, clinical, and demographic data was limited. Age at onset was available for five individuals and ranged from 20 to 43 years, whereas sex was reported for six individuals (Supplementary Table 4).

### 3.2 Genetic landscape of CH EOPD

Across the 98 included studies, 304 cases were reported as CH, encompassing 184 unique variants across 11 genes. *PRKN* accounted for the most cases (*n* = 241), followed by *PINK1* (*n* = 16) and *VPS13C* (*n* = 11), whereas the remaining genes were represented by smaller numbers of individuals (Supplementary Table 1). Most single nucleotide variants (SNVs; *n* = 127) and short indels were nonsynonymous (*n* = 71), while structural variants (SVs; *n* = 57) mainly comprised exon-level deletions and duplications. Gene-specific variant counts and annotations are provided in Figure 2 and Supplementary Tables 1–3.

**Figure 2:**
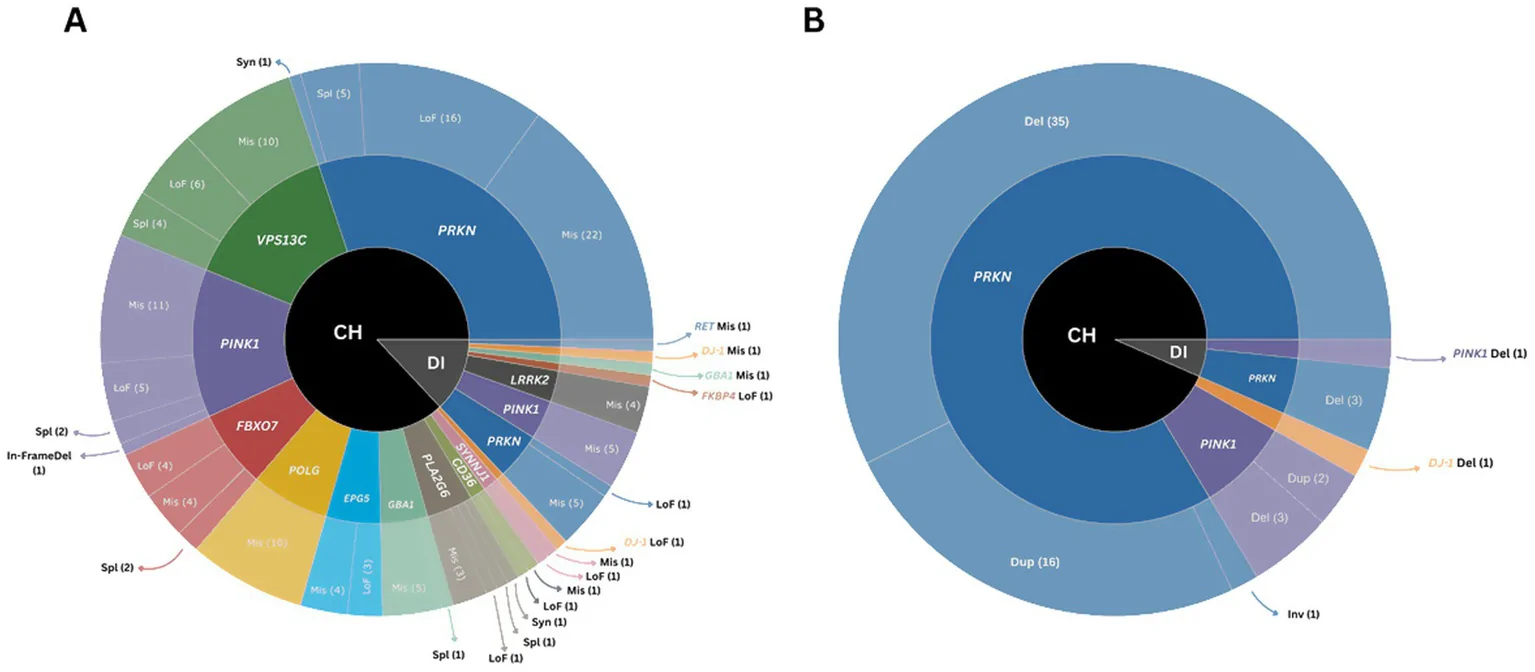
Genetic architecture across compound heterozygous (CH) and digenic inheritance (DI) early-onset Parkinson’s disease (EOPD) cases. Distribution of genes and variant molecular consequences among reported CH and DI cases. Sunburst plots depict the numbers of unique single-nucleotide variants (SNVs; **A**) and structural variants (SVs; **B**) identified across genes. The inner ring represents genes, whereas the outer ring shows variant molecular consequences. For SNVs, missense variants were the most common consequence, followed by loss-of-function and splice-site variants. For SVs, exon-level deletions predominated, particularly in *PRKN*. Numbers in parentheses indicate variant counts.

#### 3.2.1 PRKN

A total of 241 individuals with CH *PRKN* variants were included, representing 96 unique variants. SV was a prominent feature of *PRKN*-related CH, with 52 exon rearrangements observed alongside 44 SNVs and indels (Supplementary Table 2 and Figure 2B). Consistent with this, most cases involved at least one SV. The most frequent variants were Ex3del (*n* = 55), p. Arg275Trp (*n* = 41), and Ex4del (*n* = 39). Overall, 47.7% of cases involved a combination of one SV and one SNV or short indel, while 37.34% carried two distinct SVs. The recurrent genotypes Ex3del/Ex4del, Ex3del/Ex2del, Ex3del/p. Asn52fs, and Ex7del/p. Val324fs were the most frequently reported combinations (Figure 3A).

**Figure 3:**
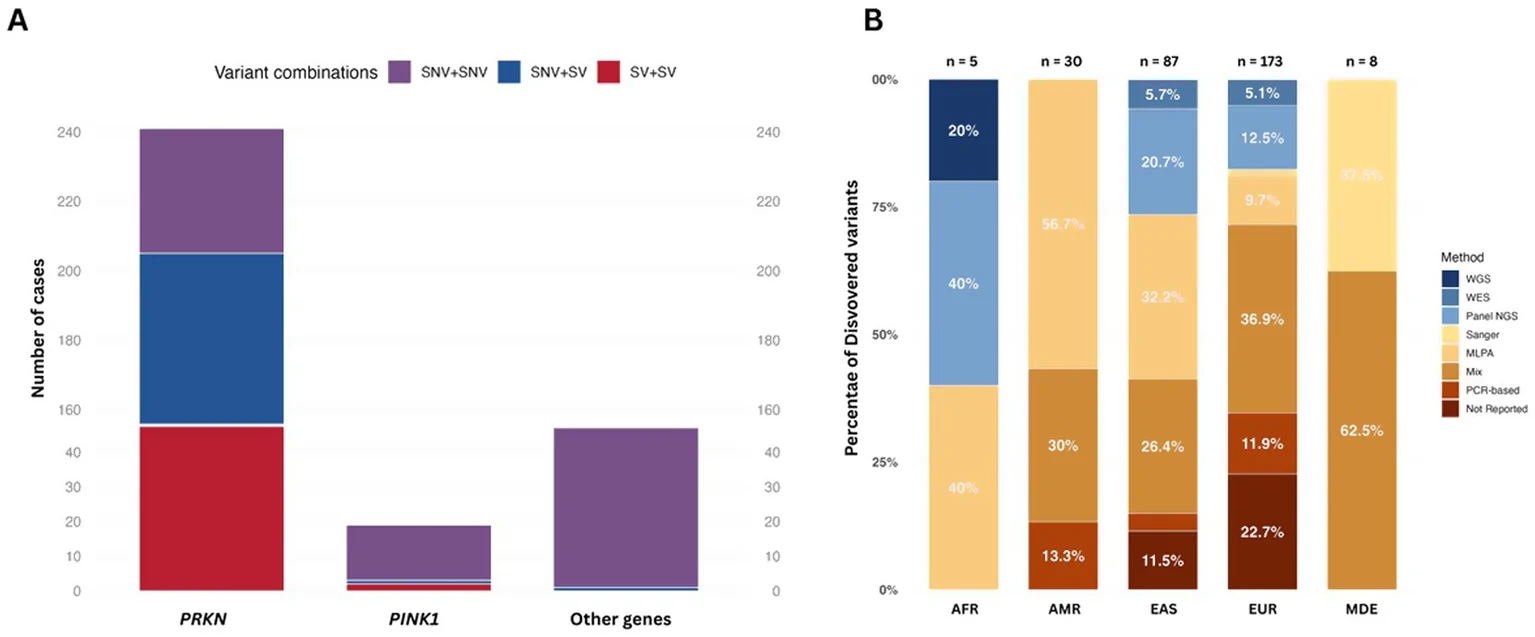
Distribution of variant type and genetic assays performed across compound heterozygous (CH) and digenic inheritance (DI) early-onset Parkinson’s disease (EOPD) cases. **(A)** Distribution of variant combinations among CH cases stratified by gene. Bars show the number of individuals carrying combinations of two SNVs/short indels, one SNV and one SV, or two SVs. *PRKN* accounted for most CH cases and was enriched for combinations involving structural variants. **(B)** Sequencing and genotyping methods reported across studies by ancestry groups (Participants with reported ancestry only). Stacked bars indicate the proportion of cases analyzed using whole-genome sequencing (WGS), whole-exome sequencing (WES), targeted gene panels, Sanger sequencing, multiplex ligation-dependent probe amplification (MLPA), PCR-based assays, or methods not reported. Additional specialized analyses, including segregation and haplotype-based approaches, were grouped as Other. Percentages represent the proportion of cases within each ancestry group analyzed using each method. Abbreviations: AFR, African; AMR, admixed American; EAS, East Asian; EUR, European; MDE, Middle Eastern.

Among the 241 individuals with *PRKN* CH, 143 (59.3%) were reported as EUR, 61 (25.3%) as EAS, 28 (11.6%) as AMR, five (2.1%) as MDE, three (1.2%) as AFR, and one (0.4%) as another ancestry category. Variant-pair recurrence across ancestry groups was limited. All 20 distinct unordered combinations observed among AMR cases were reported only in AMR individuals within this dataset (20/20, 100%). The corresponding proportions were 76/83 (91.6%) in EUR, 31/37 (83.8%) in EAS, 3/4 (75.0%) in MDE, and 2/3 (66.7%) in AFR cases. Examples included Ex7del/p. Val324fs in Italian cases; Ex12del/Ex9del and Ex4del/Ex9del in Mexican cases; c.619-1G > C/Ex1–2del, Ex2del/p. Gly284Arg, and Ex2del/Ex3del in Chinese cases; and Ex2dup/Ex9del and p. Gly139Valfs*38/p. Pro113Thrfs*51 in South African cases. Given the sparse and uneven case-report-derived evidence, these patterns describe combinations observed only within particular ancestry groups in the assembled literature and should not be interpreted as evidence of population-specific variation. Additional ancestry-specific combinations are provided in Supplementary Table 1.

#### 3.2.2 PINK1

A total of 16 individuals with CH *PINK1* variants were included, representing 24 unique variants. In contrast to *PRKN,* SVs were less prominent, and most cases involved two SNVs or short indels. Five exon rearrangements were observed, along with 19 SNVs and indels. Overall, 81.25% of cases carried two SNVs, while 10.52% of individuals involved two distinct SVs, and a combination of one SV with one SNV or short indel was observed in 6.25%. The most frequent variant was p. Arg492Ter (*n* = 5), while recurrent genotypes included occurrences of p. Arg492Ter in combination with p. Gln239Ter and p. Leu369Pro (*n* = 2, each). All of these recurrent genotypes were reported in individuals of EUR ancestry (Supplementary Tables 1, 2).

#### 3.2.3 Other genes

Aside from *PRKN* and *PINK1*, 47 individuals with CH variants were reported across several additional PD-associated genes. *VPS13C* was the most commonly represented gene in this group, followed by *FBXO7* and *PLA2G6*. Overall, 65 unique variants were identified, including a single SV in *PARK7* (Ex6del). Among SNVs and short indels, most were non-synonymous variants (*n* = 38), followed by other SNV types (*n* = 27). The most frequent variants were p. Asn370Ser in *GBA1* (*n* = 4) and p. Asp331Tyr in *PLA2G6* (*n* = 4). The single *PARK7* case identified in this review carried an Ex6 deletion in combination with p. Asn81fs. The most frequent genotype was the combination of p. Gly39Arg and p. Leu280Tyrfs*11 in *FBXO7* (*n* = 3), followed by two occurrences each of combinations in *EPG5, FBXO7, GBA1, PLA2G6, POLG, SYNJ1* and *VPS13C*. All remaining combinations were reported just once (Supplementary Tables 1, 2).

### 3.3 Gene combinations in reported DI EOPD

We identified 16 individuals with reported digenic PD, harboring 24 unique variants across seven genes: *PRKN*, *LRRK2*, *PINK1*, *PARK7*, *RET*, *GBA1*, and *FKBP4*. Eight individuals carried an *LRRK2*-associated combination, while the remaining eight carried combinations that did not involve *LRRK2*. The most frequently observed gene combinations were *LRRK2*–*PRKN* (*n* = 6), followed by *PINK1*–*PRKN* (*n* = 5) and *PARK7*–*PINK1* (*n* = 2). Although *LRRK2* is classically associated with autosomal-dominant PD, several studies have reported its co-occurrence with variants in other PD-associated genes and have interpreted these as digenic cases. These may reflect *LRRK2*-driven disease with additional modifying variants; however, these were retained in accordance with our operational inclusion criteria. Their inclusion denotes source-reported DI and does not constitute independent confirmation of digenic causality.

Among the 24 variants, 19 were SNVs or short indels. Five SVs were identified, comprising exon deletions in *PRKN* and a deletion of exon 1 in *PINK1* (Supplementary Tables 4, 5). The eight *LRRK2*-associated cases comprised 12 unique variants, including 10 SNVs or small indels and two structural variants. Six individuals harbored *LRRK2*–*PRKN* combinations, each representing a unique variant configuration. One individual carried *LRRK2* p. Gly2019Ser together with two *PRKN* structural variants (Ex3–4del and Ex9–10del). *LRRK2* p. Gly2019Ser was also reported in combination with *PRKN* p. Met192Val or p. Ala398Thr, whereas *LRRK2* p. Arg1441Gly co-occurred with *PRKN* p. Asn52fs or p. Met192Val. The remaining two individuals involved *LRRK2* p. Met262Val with *PINK1* p. Ala383Thr and *LRRK2* p. Gly2019Ser with *GBA1* p. Asn409Ser. Ancestry was reported for seven of the eight *LRRK2*-associated cases (one missing): five were EUR, one was AMR, and one was AFR (Supplementary Tables 4, 5).

After exclusion of all *LRRK2*-associated cases, the *LRRK2*-excluded sensitivity subgroup comprised eight individuals harboring 13 unique variants, including 10 SNVs or small indels and three structural variants. The sequence variants included nine nonsynonymous SNVs and one non-frameshift deletion, whereas all three structural variants were exon-level deletions. Two individuals of admixed American ancestry, both reported in a Mexican family, carried a *PINK1* exon 1 deletion in combination with distinct PRKN structural variants (Ex9–12del and Ex7del, respectively). The *PARK7–PINK1* combination was identified in two affected sisters from a Chinese family carrying *PARK7* p. Ala39Ser and *PINK1* p. Pro399Leu. The remaining individual carried *FKBP4* p. Arg144His in combination with *RET* p. Glu422del (Supplementary Tables 4, 5).

Variants with uncertain or conflicting clinical classifications were retained when the source publication reported the corresponding configuration as digenic and the variant did not meet the predefined exclusion criteria. Their inclusion should not be interpreted as independent confirmation of variant pathogenicity or of a causal digenic mechanism.

### 3.4 Genetic assays

Genetic assays varied across studies and frequently involved complementary approaches. MLPA (*n* = 64) and targeted NGS panels (*n* = 42) were the most commonly reported assays across all populations, while PCR-based assays were restricted to AMR, EAS and EUR cases; Sanger sequencing was used exclusively for EUR and MDE populations, and WES and WGS were used less frequently overall. MLPA was often combined with sequencing-based methods to detect exon-level rearrangements, particularly in *PRKN* and *PINK1* (*n* = 102). Additional specialized analyses, including segregation and haplotype-based approaches, were grouped as “Other” (Supplementary Table 1 and Figure 3B).

### 3.5 Clinical features and genotype–phenotype associations

#### 3.5.1 Motor and non-motor features in CH EOPD

Motor and non-motor features were reported across 59 CH studies. Motor manifestations were dominated by the cardinal features of PD, with rigidity, bradykinesia, and tremor being the most frequently reported symptoms across several genetic subtypes, including *PRKN, PINK1, PLA2G6, VPS13C*, and *FBXO7*. Gait disturbance and postural instability were also frequently described, whereas dystonia, dyskinesia, akinesia, hypomimia, hypophonia, and freezing of gait were reported less frequently. Motor complications related to dopaminergic therapy were documented in a subset of individuals, with wearing-off phenomena reported in 11 cases across several genetic subtypes (Table 2).

Non-motor features were heterogeneous and less consistently reported. Among autonomic manifestations, constipation was the most frequent, followed by olfactory impairment, predominantly reported in individuals carrying *PRKN* and *EPG5* variants. Psychiatric symptoms, particularly depression, anxiety, and psychosis, were among the most frequently described manifestations, mainly in individuals carrying *PRKN, VPS13C,* and *PINK1*. Cognitive impairment was reported along with dementia across *PRKN, DJ-1, EPG5,* and *PLA2G6*. Sleep disturbance was reported in 14 cases across multiple genetic subtypes (Table 2).

#### 3.5.2 Motor severity and neuropsychological measures in CH EOPD

Motor severity was reported across 34 studies, with substantial variability across available scales. MDS-UPDRS scores ranged from 11 to 92 and UPDRS III from 0 to 62, indicating substantial heterogeneity in motor impairment. Hoehn and Yahr stages spanned from early to advanced disease (1–5). These measures were reported across multiple genetic subtypes, with no evidence of a consistent genotype–severity association (Table 2).

Cognitive and neuropsychological assessments were reported across 20 articles. Among individuals with available MMSE data, scores ranged from normal to mildly impaired, whereas available MoCA assessments more often fell within ranges consistent with cognitive impairment. Moderate-to-severe impairment was reported in a small number of individuals with dementia. HAMD and HAMA scores, when available, were generally within the mild-to-moderate symptom range (Table 2).

#### 3.5.3 Pharmacological therapies in CH EOPD

Treatment approaches were reported in 37 studies and were dominated by dopaminergic therapies. Levodopa-based regimens were the most frequently used treatment, spanning a wide range of genetic subtypes, while dopamine agonists and related agents were used less commonly. Adjunctive therapies, including amantadine, anticholinergics, and MAO-B inhibitors, were reported in a minority of cases, particularly among individuals with *PRKN*. Treatments targeting neuropsychiatric or cognitive symptoms were rarely reported. Overall, treatment strategies appeared to be guided by clinical phenotype rather than genotype (Table 3).

#### 3.5.4 Neuroimaging and ancillary investigations in CH EOPD

Neuroimaging and ancillary investigations were reported in 21 articles. Structural MRI findings were heterogeneous. Normal imaging was reported in several cases, particularly among PRKN-associated cases, whereas reported abnormalities included cortical atrophy, with frontal, temporal, and parietal involvement, sometimes accompanied by cerebellar atrophy. Additional findings included basal ganglia signal abnormalities consistent with iron deposition and substantia nigra changes suggestive of nigrostriatal degeneration. Advanced MRI techniques identified loss of the nigral swallow-tail sign and reduced neuromelanin signal in selected cases.

Among individuals with available dopaminergic imaging, reduced striatal dopamine transporter uptake was reported across several genetic subtypes, including *PRKN, PINK1, VPS13C,* and *POLG,* often with bilateral or putaminal-predominant involvement. Similarly, ^18^F-DOPA PET and related modalities showed reduced uptake in the putamen and caudate nucleus in reported cases. FDG-PET findings were more heterogeneous, with cortical hypometabolism reported across frontal, temporal, parietal, and occipital regions, together with occasional subcortical abnormalities and relative hypermetabolism in selected regions. Neuropathological findings were available only for a limited number of *VPS13C* cases and included cortical atrophy, severe substantia nigra neuronal loss, and mixed proteinopathies (Table 3).

#### 3.5.5 Clinical features in cases reported as DI

Clinical features were reported across 16 individuals classified as having DI in the source publications and were dominated by cardinal PD symptoms. Age at onset was available for only five individuals: One of eight *LRRK2*-associated cases (43 years) and four of eight individuals in the *LRRK2-*excluded subgroup (20–42 years). Given the extent of missing data, age at onset could not be meaningfully compared between the two subgroups. Among cases with available data, females were more frequently reported than males. When treatment data were available, most individuals improved with levodopa-based therapy, whereas dopamine agonists and adjunctive antiparkinsonian agents were used less frequently.

Clinical reporting was heterogeneous, with limited information on motor severity, progression, non-motor burden, and longitudinal outcomes, thereby limiting robust comparisons across subgroups of reported DI cases. Additional motor manifestations were reported in selected cases and appeared to vary with reported gene combinations. Dystonia, facial dyskinesias, and dysarthria were mainly reported in individuals carrying *PINK1–PRKN* combinations, whereas freezing of gait and postural instability were reported in isolated *LRRK2–PRKN* and *PINK1–PRKN* cases.

Non-motor features were less consistently documented but were most frequently reported in *PINK1–PRKN* cases, including depression, anxiety, psychosis, constipation, sleep disturbance, and cognitive impairment. In contrast, *LRRK2–PRKN* cases were more often described as presenting with predominantly motor phenotypes. When dopaminergic imaging was reported, reduced striatal tracer uptake was observed, consistent with presynaptic nigrostriatal dysfunction.

## 4 Discussion

In this systematic review of 106 studies comprising 304 reported compound heterozygous (CH) and 16 cases reported as digenic inheritance (DI) early-onset Parkinson disease (EOPD) cases, we provide the first unified synthesis to date of these literature-reported categories of EOPD genetic findings. CH represented conventional biallelic recessive inheritance and was largely driven by *PRKN* structural variation. In contrast, configurations reported as DI were rare, genetically heterogeneous, and showed limited recurrence at the variant level. Clinically, both models converged toward a core Parkinsonian syndrome, with variable motor and non-motor involvement but no consistent genotype-specific clinical profile. Together, these findings refine the genetic and clinical architecture of EOPD and illustrate the range of rare variant combinations that have been proposed in both distinct mechanisms to contribute to disease susceptibility and phenotypic expression.

Our findings indicate a markedly asymmetric genetic architecture of CH EOPD, with clear predominance of *PRKN*. Structural variation was a major driver of this pattern, with most *PRKN* CH cases harboring at least one exon-level rearrangement, most commonly single- or multi-exon deletions. This enrichment is consistent with the known mutational susceptibility of the *PRKN* locus and likely contributes to its high representation among reported CH cases (Seike et al., 2021). The large number of *PRKN* cases also enabled the identification of recurrent genotypes, frequently involving exon deletions in combination with previously reported disease-associated *PRKN* variants, including p. Arg275Trp and p. Gln34ArgfsTer5 (Milanowski et al., 2021). Notably, many CH cases were identified using MLPA, usually in combination with sequencing-based approaches. Differences in access to and use of MLPA across studies and populations may therefore have influenced the observed distribution of exon-level rearrangements, particularly at the *PRKN* locus. Although MLPA remains effective for detecting exon-level rearrangements, its limited resolution may not fully characterize complex structural variants or determine phase and allelic configuration (Fant et al., 2025). These limitations are particularly relevant given the potential contribution of underexplored non-canonical variant classes, including short tandem repeat expansions. For example, one additional reported CH case involved biallelic pathogenic *RFC1* repeat expansions but could not be systematically analyzed because of insufficient variant-level annotation (Hirano et al., 2025). Future studies integrating WGS with long-read sequencing or optical mapping could improve breakpoint and orientation resolution, resolve complex rearrangements and phase, and thereby strengthen confirmation of biallelic CH and interpretation of SV-containing configurations reported as DI. Long-read approaches may also improve detection of underexplored variant classes such as repeat expansions.

In contrast to the *PRKN*-driven architecture observed in CH EOPD, DI cases were rare and genetically heterogeneous. Across 16 individuals, we identified 24 variants spanning seven genes, with most reported combinations involving genes previously associated with PD, particularly *PRKN*, *LRRK2*, and *PINK1*. Although this pattern is biologically plausible given the known contribution of these genes to monogenic and EOPD, it should be interpreted cautiously because it may also reflect gene-selection bias in diagnostic testing and case reporting. The most frequent gene combinations were *LRRK2*–*PRKN* and *PINK1*–*PRKN*; however, most variant pairs were unique and showed limited recurrence beyond the gene level. This lack of recurrent variant-level patterns suggests that reported DI cases remain difficult to distinguish from coincidental co-occurrence of rare variants or monogenic disease modified by additional variants. Our counts therefore describe configurations reported as DI in the literature rather than independently confirmed causal categories.

In the reviewed DI cases, combinations involving *LRRK2* required particular caution in interpretation. *LRRK2* is classically associated with autosomal dominant PD (Dächsel et al., 2006; Lesage et al., 2006), and recurrent pathogenic variants such as p. Gly2019Ser have incomplete penetrance (Dächsel et al., 2006; Lesage et al., 2006). Therefore, the co-occurrence of *LRRK2* variants with heterozygous variants in other PD-associated genes may represent true biological interaction in some cases but may also reflect *LRRK2*-driven disease with additional modifying variants rather than bona fide DI. Although experimental evidence supports biological interactions between selected PD genes, including PINK1 and DJ-1 in oxidative stress pathways (Tang et al., 2006), most reported DI cases lack the segregation analyses, replication across unrelated families, and functional validation that would be needed to support a causal digenic model. Because this review did not perform independent case-level causal adjudication, these alternative interpretations could not be resolved or ranked consistently for individual cases.

Beyond genetic architecture, the reviewed cases also highlighted substantial clinical heterogeneity across CH and DI EOPD. In CH cases, clinical presentation was centered on the core Parkinsonian syndrome, whereas age at onset, motor severity, and non-motor burden varied widely between and within genetic subtypes (Alcalay et al., 2011; Shen et al., 2019; Gao et al., 2020; Li et al., 2020; Yu et al., 2021). *PRKN*-associated cases generally aligned with prior reports of earlier onset, slower progression, favorable levodopa responsiveness, and frequent dystonia or treatment-related dyskinesia. In contrast, less common subtypes, particularly *PLA2G6* and *VPS13C*, more often showed cognitive impairment, psychiatric manifestations, pyramidal signs, or structural imaging abnormalities, consistent with a broader neurodegenerative phenotype (Lesage et al., 2016; Smolders et al., 2021; Monfrini et al., 2022; Menon et al., 2024).

Among DI cases, clinical interpretation was more limited because of the small number of reports and incomplete phenotypic characterization. *PINK1–PRKN* combinations appeared to show broader phenotypic expression in some reports (Funayama et al., 2008; Monroy-Jaramillo et al., 2014; Hayashida et al., 2021; Landoulsi et al., 2023), whereas *LRRK2–PRKN* cases were more often described as resembling established *LRRK2*-associated presentations (Dächsel et al., 2006; Lesage et al., 2006; Landoulsi et al., 2023). Overall, the available clinical data did not support robust genotype-specific profiles but instead suggested broad phenotypic variability across both CH and DI models. This variability may have practical implications for clinical recognition, as mixed or atypical genetic architectures could be underrecognised in patients who do not fit a classic monogenic presentation. In addition, the substantial non-motor burden reported across cases, including psychiatric symptoms, cognitive impairment, autonomic dysfunction, and sleep disturbance, highlights the importance of systematic clinical assessment beyond motor features in EOPD.

Neuroimaging and ancillary investigations provided additional evidence of both convergence and divergence across genetic subtypes. Structural imaging was frequently normal in *PRKN*-related CH cases, whereas less common subtypes, such as *PLA2G6* and *VPS13C*, were more often associated with cortical or cerebellar atrophy, basal ganglia signal abnormalities suggestive of iron deposition, or more diffuse structural changes (Fang et al., 2019; Shen et al., 2019; Smolders et al., 2021; Monfrini et al., 2022). In contrast, dopaminergic imaging, including DaTSCAN, DAT-SPECT, and ^18^F-DOPA PET, showed a more consistent pattern across genes, commonly demonstrating reduced striatal uptake with putaminal predominance, consistent with shared presynaptic nigrostriatal dysfunction (Rango et al., 2013; Huang et al., 2019; Stark et al., 2019; Gao et al., 2020; Daida et al., 2023; Dulski et al., 2024). Similar findings were reported in a limited number of DI cases with functional imaging. Overall, the available imaging and pathological data suggest two broad patterns: a relatively selective nigrostriatal profile in *PRKN*-predominant disease and a more diffuse multisystem profile in rarer subtypes such as *PLA2G6* and *VPS13C*.

Beyond these observations, our review highlights differences in how CH and reported DI configurations are captured by existing PD genetic resources. Gene-centered resources such as MDSGene are well suited to documenting within-gene biallelic variation but are less suited to examining cross-gene configurations reported as DI. Our variant-centric approach permitted harmonized description of both categories while maintaining their conceptual distinction. It confirmed the predominance of conventional PRKN-associated recessive CH, whereas the evidence supporting causal DI remained sparse and uncertain.

This study has several strengths. It provides the first systematic and comprehensive synthesis of reported CH and DI EOPD cases, integrating both genetic and clinical data across a large number of studies. In addition, our detailed variant-level curation and harmonization enabled standardization of heterogeneous reports and cross-gene comparisons of within-gene CH and cross-gene configurations reported as DI, providing a level of resolution not achievable through gene-specific approaches alone. Lastly, the integration of available clinical data with genetic findings also enabled a preliminary assessment of genotype–phenotype associations across these models.

At the same time, several limitations should be acknowledged. First, the study was constrained by substantial heterogeneity in genetic reporting across the literature, including inconsistent variant nomenclature, transcript selection, and limited characterization of structural variants. Although extensive manual curation was undertaken, ambiguities inherent to the original reports could not be fully resolved. Second, clinical data were incomplete and inconsistently reported, limiting assessment of genotype–phenotype associations. Consequently, no inferential statistical tests were performed to compare age at onset or clinical-feature frequencies across gene groups, and the observed patterns should be interpreted as descriptive. Related individuals were retained when separate individual-level data were available but should not be interpreted as independent replication. Third, ancestry patterns among published cases may reflect family clustering, referral and testing practices, publication bias, and the ancestral composition of the available literature. The predominance of EUR studies further limits generalizability, and the reported ancestry distributions should not be interpreted as population-level differences. Addressing these disparities will require more ancestrally diverse cohorts and equitable access to comprehensive genetic testing, including methods capable of detecting both sequence and structural variation, to ensure that advances in genetic diagnosis benefit populations globally. Finally, cases reported as DI were few, and several included variants had uncertain, conflicting, or unavailable clinical classifications. The source publications also frequently lacked segregation analyses, functional validation, replication in unrelated families, or sufficient information to determine whether either allele could independently explain the phenotype. Because we did not undertake independent ACMG/AMP classification or case-level causal adjudication, the contribution of individual variants and the distinction between causal DI, modifier effects, and coincidental co-occurrence remain unresolved.

### 5 Conclusion

This systematic review provides an integrated synthesis of published CH and reported DI cases in EOPD as of mid-2026. CH, largely driven by *PRKN* exon-level structural variation, represents a conventional biallelic autosomal-recessive mechanism. In contrast, reported DI configurations were rare and heterogeneous and generally lacked the evidence required to distinguish causal cross-gene effects from modifier relationships or coincidental co-occurrence. Despite substantial genetic heterogeneity, reported cases converged clinically toward a shared Parkinsonian phenotype and common patterns of nigrostriatal dysfunction, suggesting that diverse and rare variant interactions may funnel into overlapping neurobiological pathways. These findings support comprehensive detection of structural and sequence variants while emphasizing the need for segregation, functional, and replication evidence before causal DI can be established.

## Data Availability

All data analyzed during this study are included in this published article and its Supplementary material. The data underlying this study were derived from previously published articles, which are cited throughout the manuscript. The custom code used for data extraction, curation, analysis, and figure generation is publicly available at: https://github.com/computational-neurogenomics/compound_het_DI_review.git.
