# Supplementary material for: Compound heterozygous and digenic inheritance in early-onset Parkinson’s disease: a systematic review
Source: Front Aging Neurosci. 2026 Oct 22;18:1930518. doi: 10.3389/fnagi.2026.1930518 (PMC13736722; doi:10.3389/fnagi.2026.1930518)
Supplement: Supplementary file 1 [file Data_Sheet_1.PDF]

Supplementary Methods

Compound heterozygous and digenic inheritance in early-onset Parkinson's disease: A Systematic Review

Contents

This file contains the following supplementary information for the manuscript:

S1. PRISMA Reporting Information
- **S1.1** Literature search
- **S1.2** Compound heterozygous search
- **S1.3** Digenic inheritance search
- **S1.4** Search yield and record flow
- **S1.5** PRISMA 2020 Checklist

S2. Study selection and eligibility
- **S2.1** Evidence synthesis and applicability of PRISMA appraisal items
- **S2.2** Limitations of the evidence and review process
- **S2.3** Availability of materials

S3. Genetic variant harmonisation and quality-control audit
- **S3.1** Scope and input data
- **S3.2** Genome assembly and sequence-variant normalisation
- **S3.3** Automated internal-consistency checks
- **S3.4** Manual review of flagged variants
- **S3.5.** Audit code and reproducibility

S1. PRISMA Reporting Information

This supplement reports the complete literature-search strategies, record-management and selection procedures, eligibility criteria, data-curation methods, and the scope of the descriptive evidence synthesis. The two searches were treated as components of one consolidated systematic-review run.

S1.1 Literature search

PubMed was searched on 8 September 2026. Both searches covered publications dated from 1 January 1900 through 8 September 2026. No additional language, article-type, or geographic restrictions were applied. Search results were exported in CSV format, combined, and deduplicated before screening.

S1.2 Compound heterozygous search

((compound heterozygous AND Parkinson's disease) OR (Parkinson*[Title/Abstract] AND ("double heterozyg*"[Title/Abstract] OR "biallelic heterozyg*"[Title/Abstract] OR "variant in trans"[Title/Abstract:~0] OR "variants in trans"[Title/Abstract:~0] OR "mutation in trans"[Title/Abstract:~0] OR "mutations in trans"[Title/Abstract:~0]))) AND 1900/01/01:2026/09/08[Date - Publication]

S1.3 Digenic inheritance search

((digenic AND Parkinson's disease) OR (Parkinson*[Title/Abstract] AND ("double heterozyg*"[Title/Abstract] OR oligogenic[Title/Abstract] OR multilocus[Title/Abstract] OR "multi-locus"[Title/Abstract] OR "modifier variant*"[Title/Abstract] OR "modifier allele*"[Title/Abstract]))) AND 1900/01/01:2026/09/08[Date - Publication]

S1.4 Search yield and record flow

The compound-heterozygous search returned 299 records and the digenic-inheritance search returned 67 records. After combining the 366 retrieved records, eight duplicates were removed, leaving 358 unique records for title and abstract screening.

Stage	***n***	***Details***
Records identified	366	CH search, n = 299; DI search, n = 67
Duplicate records removed	8	Removed before screening
Unique records screened	358	Title and abstract screening
Records excluded	186	Excluded after title and abstract screening
Reports sought for retrieval	172	All were retrieved
Reports not retrieved	0	-
Reports assessed for eligibility	172	Full-text and variant-level assessment
Reports excluded	66	Reasons reported below
Reports included	106	CH, n = 98; DI, n = 9; one report contributed to both categories

PRISMA 2020 Checklist

Section and Topic	Item #	Checklist item	Location where item is reported
**TITLE**			
Title	1	Identify the report as a systematic review.	Title
**ABSTRACT**			
Abstract	2	See the PRISMA 2020 for Abstracts checklist.	Abstract
**INTRODUCTION**			
Rationale	3	Describe the rationale for the review in the context of existing knowledge.	Section1, paragraphs 1-3
Objectives	4	Provide an explicit statement of the objective(s) or question(s) the review addresses.	Abstract (Introduction); Section 1, paragraph 4.
**METHODS**			
Eligibility criteria	5	Specify the inclusion and exclusion criteria for the review and how studies were grouped for the syntheses.	Section 2.1.1; Supplementary Methods S2.
Information sources	6	Specify all databases, registers, websites, organisations, reference lists and other sources searched or consulted to identify studies. Specify the date when each source was last searched or consulted.	Section 2.1; Supplementary Methods S1.1.
Search strategy	7	Present the full search strategies for all databases, registers and websites, including any filters and limits used.	Supplementary Methods S1.2-S1.3.
Selection process	8	Specify the methods used to decide whether a study met the inclusion criteria of the review, including how many reviewers screened each record and each report retrieved, whether they worked independently, and if applicable, details of automation tools used in the process.	Sections 2.1 and 2.1.1; Supplementary Methods S2.
Data collection process	9	Specify the methods used to collect data from reports, including how many reviewers collected data from each report, whether they worked independently, any processes for obtaining or confirming data from study investigators, and if applicable, details of automation tools used in the process.	Section 2.1.2.
Data items	10a	List and define all outcomes for which data were sought. Specify whether all results that were compatible with each outcome domain in each study were sought (e.g. for all measures, time points, analyses), and if not, the methods used to decide which results to collect.	Section 2.1.2; Tables 2-3.
	10b	List and define all other variables for which data were sought (e.g. participant and intervention characteristics, funding sources). Describe any assumptions made about any missing or unclear information.	Sections 2.1.2 and 2.2; Supplementary Methods S3.1; Supplementary Tables 1-5.
Study risk of bias assessment	11	Specify the methods used to assess risk of bias in the included studies, including details of the tool(s) used, how many reviewers assessed each study and whether they worked independently, and if applicable, details of automation tools used in the process.	N/A - no formal study-level risk-of-bias assessment was performed; Supplementary Methods S2.1-S2.2.
Effect measures	12	Specify for each outcome the effect measure(s) (e.g. risk ratio, mean difference) used in the synthesis or presentation of results.	N/A - no

PRISMA

PRISMA 2020 Checklist

Section and Topic	Item #	Checklist item	Location where item is reported
			comparative effect measure was calculated; descriptive counts and proportions only (Supplementary Methods S2.1).
Synthesis methods	13a	Describe the processes used to decide which studies were eligible for each synthesis (e.g. tabulating the study intervention characteristics and comparing against the planned groups for each synthesis (item #5)).	Sections 2.1.1-2.2; Supplementary Methods S2.
	13b	Describe any methods required to prepare the data for presentation or synthesis, such as handling of missing summary statistics, or data conversions.	Sections 2.1.2 and 2.2; Supplementary Methods S3.1-S3.4; Supplementary Tables 1-5.
	13c	Describe any methods used to tabulate or visually display results of individual studies and syntheses.	Section 2.2; Supplementary Methods S2.1; Tables 1-3; Figures 1-3 and legends.
	13d	Describe any methods used to synthesize results and provide a rationale for the choice(s). If meta-analysis was performed, describe the model(s), method(s) to identify the presence and extent of statistical heterogeneity, and software package(s) used.	Sections 2.1.2 and 2.2; Supplementary Methods S2.1 (descriptive case-level synthesis using R v4.4.0 and custom scripts).
	13e	Describe any methods used to explore possible causes of heterogeneity among study results (e.g. subgroup analysis, meta-regression).	N/A - no formal heterogeneity analysis was performed; descriptive stratification is reported in Sections 3.2-3.5.
	13f	Describe any sensitivity analyses conducted to assess robustness of the synthesized results.	Sections 3.3 and 3.5.5 (descriptive LRRK2-excluded subgroup analysis).
Reporting bias assessment	14	Describe any methods used to assess risk of bias due to missing results in a synthesis (arising from reporting biases).	N/A - no formal reporting-bias assessment was performed; Supplementary Methods S2.1-

Section and Topic	Item #	Checklist item	Location where item is reported
			S2.2.
Certainty assessment	15	Describe any methods used to assess certainty (or confidence) in the body of evidence for an outcome.	N/A - no certainty-of-evidence or GRADE assessment was performed; Supplementary Methods S2.1-S2.2.
**RESULTS**			
Study selection	16a	Describe the results of the search and selection process, from the number of records identified in the search to the number of studies included in the review, ideally using a flow diagram.	Section 3.1; Figure 1; Supplementary Methods S1.4.
	16b	Cite studies that might appear to meet the inclusion criteria, but which were excluded, and explain why they were excluded.	Not reported for individual excluded studies; aggregate exclusion reasons and counts are provided in Supplementary Methods S2 and Figure 1.
Study characteristics	17	Cite each included study and present its characteristics.	Section 3.1; Supplementary Tables 1 and 4; Tables 1-3.
Risk of bias in studies	18	Present assessments of risk of bias for each included study.	N/A - no formal study-level risk-of-bias assessment was performed; Supplementary Methods S2.1-S2.2.
Results of individual studies	19	For all outcomes, present, for each study: (a) summary statistics for each group (where appropriate) and (b) an effect estimate and its precision (e.g. confidence/credible interval), ideally using structured tables or plots.	Supplementary Tables 1-5 and Tables 1-3 (descriptive case-level data); N/A for effect estimates and precision.
Results of syntheses	20a	For each synthesis, briefly summarise the characteristics and risk of bias among contributing studies.	Sections 3.1-3.5; Tables 1-3; Supplementary Tables 1-5. Risk-of-bias assessment: N/A.

PRISMA 2020 Checklist

Section and Topic	Item #	Checklist item	Location where item is reported
	20b	Present results of all statistical syntheses conducted. If meta-analysis was done, present for each the summary estimate and its precision (e.g. confidence/credible interval) and measures of statistical heterogeneity. If comparing groups, describe the direction of the effect.	N/A - no statistical synthesis or meta-analysis was performed; descriptive results are reported in Sections 3.2-3.5.
	20c	Present results of all investigations of possible causes of heterogeneity among study results.	N/A - no formal investigation of statistical heterogeneity was performed; descriptive subgroup patterns are reported in Sections 3.2-3.5.
	20d	Present results of all sensitivity analyses conducted to assess the robustness of the synthesized results.	Sections 3.3 and 3.5.5 (LRRK2-excluded descriptive sensitivity subgroup).
Reporting biases	21	Present assessments of risk of bias due to missing results (arising from reporting biases) for each synthesis assessed.	N/A - no formal reporting-bias assessment was performed; Supplementary Methods S2.1-S2.2.
Certainty of evidence	22	Present assessments of certainty (or confidence) in the body of evidence for each outcome assessed.	N/A - no certainty-of-evidence or GRADE assessment was performed; Supplementary Methods S2.1-S2.2.
**DISCUSSION**			
Discussion	23a	Provide a general interpretation of the results in the context of other evidence.	Section 4, Discussion.
	23b	Discuss any limitations of the evidence included in the review.	Section 4, limitations paragraph; Supplementary Methods S2.2.
	23c	Discuss any limitations of the review processes used.	Section 4, limitations

PRISMA 2020 Checklist

Section and Topic	Item #	Checklist item	Location where item is reported
			paragraph; Supplementary Methods S2.2.
	23d	Discuss implications of the results for practice, policy, and future research.	Sections 4 and 4.1.
**OTHER INFORMATION**			
Registration and protocol	24a	Provide registration information for the review, including register name and registration number, or state that the review was not registered.	N/A - review registration was not reported.
	24b	Indicate where the review protocol can be accessed, or state that a protocol was not prepared.	N/A - no review protocol was reported.
	24c	Describe and explain any amendments to information provided at registration or in the protocol.	N/A - no registration or protocol amendments were applicable.
Support	25	Describe sources of financial or non-financial support for the review, and the role of the funders or sponsors in the review.	Acknowledgements (no specific study funding; author support disclosed; funder role not reported).
Competing interests	26	Declare any competing interests of review authors.	Competing interests.
Availability of data, code and other materials	27	Report which of the following are publicly available and where they can be found: template data collection forms; data extracted from included studies; data used for all analyses; analytic code; any other materials used in the review.	Data availability; Code availability; Supplementary Methods S2.3.

From: Page MJ, McKenzie JE, Bossuyt PM, Boutron I, Hoffmann TC, Mulrow CD, et al. The PRISMA 2020 statement: an updated guideline for reporting systematic reviews. BMJ 2021;372:n71. doi: 10.1136/bmj.n71. This work is licensed under CC BY 4.0. To view a copy of this license, visit https://creativecommons.org/licenses/by/4.0/

S2. Study selection and eligibility

Titles and abstracts were assessed first, followed by full-text assessment. Two reviewers independently assessed records and reports, and disagreements were resolved by consensus. Duplicate records were removed before screening. Reports that described overlapping cohorts or datasets were assessed at full text so that duplicate case-level information was not retained.

Studies were eligible when they reported individuals with Parkinson's disease classified as compound heterozygous or digenic inheritance and provided sufficient individual-level genetic information to identify the relevant variants. Variants were required to be classified as pathogenic or likely pathogenic or to have additional evidence supporting pathogenicity.

Studies were excluded when they lacked individual-level genetic data; reported no eligible compound-heterozygous or digenic case; described a non-Parkinson's disease phenotype; provided insufficient variant detail; duplicated or substantially overlapped a previously represented cohort or dataset; described the inheritance model only as unclear, secondary, or putative; reported variants that did not meet the pathogenicity criteria; or lacked sufficient information for variant-level assessment.

Reason for exclusion after full-text assessment	***n***
No individual-level genetic data	12
No eligible CH or DI case identified	18
Non-PD phenotype	6
Insufficient variant detail	5
Overlapping cohort or duplicate dataset	4
CH/DI finding unclear, secondary, or putative	15
Variants did not meet pathogenicity criteria	4
Insufficient information for variant-level assessment	2
Total	66

S2.1 Evidence synthesis and applicability of PRISMA appraisal items

The review used a descriptive, case-level synthesis. Frequencies and proportions were summarised by gene, variant type, ancestry, inheritance model, and clinical domain using R v4.4.0 and custom scripts. No comparative effect measure, pooled effect estimate, or meta-analysis was calculated because the included literature consisted primarily of heterogeneous case reports, case series, and descriptive genetic studies with incompletely and

inconsistently reported phenotypes. No sensitivity analysis was undertaken because there was no pooled statistical estimate whose robustness could be tested.

Because this review was designed as a descriptive synthesis of published individual-level genetic and phenotypic data rather than an estimation of comparative effects, no formal study-level risk-of-bias, reporting-bias, or certainty-of-evidence assessment was performed.

A GRADE assessment was not performed. GRADE is principally intended to rate certainty in bodies of evidence supporting defined outcome effects. The present review catalogued reported genotypes and phenotypes and did not estimate an intervention effect, exposure association, diagnostic-accuracy measure, or other comparable pooled outcome. A formal GRADE rating would therefore not have corresponded to the review's descriptive research objective.

S2.2 Limitations of the evidence and review process

The evidence was not formally graded for risk of bias or certainty, and the findings therefore remain dependent on the completeness, methodological quality, and selective reporting of the source literature.

The review was limited to PubMed and may have missed eligible reports indexed only in other bibliographic databases or present in the grey literature. The search depended on compound-heterozygous, digenic, oligogenic, multilocus, and modifier terminology in titles, abstracts, or indexing; eligible cases described using different terminology may therefore have been missed. Although the search strategies were designed to capture variation in terminology, completeness cannot be guaranteed.

The source literature was heterogeneous in study design, ascertainment, genetic assays, variant nomenclature, transcript selection, evidence for phase, segregation analysis, functional validation, and phenotype reporting. Publication and selective-reporting biases may favour unusual, severe, or apparently novel genetic findings. These limitations are particularly relevant to digenic cases, for which classification frequently relied on the interpretation of the source authors and could not always distinguish true biological interaction from modifier effects or coincidental variant co-occurrence.

S2.3 Availability of materials

The extracted and curated data used in the analyses are provided in the article and its Supplementary Information files. The underlying evidence derives from the published articles cited in the manuscript. Custom code used for data extraction, curation, analysis, and figure generation is available at https://github.com/computational-neurogenomics/compound_het_DI_review.git.

S3 Genetic variant harmonisation and quality-control audit

S3.1 Scope and input data

A systematic quality-control audit was undertaken across the case-level and variant-level datasets used in the compound-heterozygous (CH) and reported digenic-inheritance (DI) analyses. The audit aimed to identify inconsistencies arising from heterogeneous source nomenclature, conversion between transcript-level and genomic representations, or automated annotation. It was not intended to independently reassess variant pathogenicity or molecularly re-diagnose individual cases.

For each case, the available source-reported information, including gene, nucleotide and protein designations, transcript, zygosity, structural-variant description, and inheritance configuration, was compared with the harmonised fields described in **Section 2.2**. **Supplementary Table 2** contains the source-reported and harmonised designations and was expanded to include the genome build, transcript information where available, annotation source, QC status, audit flag, supporting cross-reference, and final resolution.

S3.2 Genome assembly and sequence-variant normalisation

Single-nucleotide variants and small insertions and deletions were represented on the GRCh38/hg38 reference assembly using chromosome, position, reference, and alternate allele fields. Indels were left-aligned and normalised against the GRCh38 reference FASTA using bcftools norm v1.20. Normalised VCF records were converted to ANNOVAR input format using convert2annovar.pl with the vcf4, allsample, and withfreq options.

Functional annotation was performed with ANNOVAR using the hg38 build and the RefGene, ClinVar 21 July 2025, and dbNSFP v4.7a databases. RefGene was used to obtain gene- and transcript-level consequences, while available ClinVar clinical significance, review status, conditions, and allele identifiers were retained. Variants described using different nomenclatures were consolidated only when they represented the same molecular event.

Structural variants were reviewed separately because genomic breakpoints were rarely reported. Exon-level deletions, duplications, and inversions were retained at the resolution provided by the original publication. Source and harmonised descriptions were checked for agreement in the affected gene, event type, and exon range.

S3.3 Automated internal-consistency checks

Automated QC checks were implemented in R v4.4.0. These checks compared the source-reported and harmonised labels with the assigned gene, transcript annotation, normalised genomic allele, ANNOVAR consequence, and chromosome–position–reference–alternate identifier. The audit flagged:

1. disagreement between reported frameshift, in-frame, or termination labels and the annotated consequence;
2. disagreement between an indel consequence and the expected reading-frame effect based on reference and alternate allele lengths;
3. discrepancies in the gene, amino-acid reference, residue position, transcript annotation, genomic coordinates, or genomic identifier;
4. disagreement between source-reported and harmonised deletion or duplication events; and
5. internally incompatible protein nomenclature, such as simultaneous synonymous and frameshift designations.

Missing genomic or transcript information was not automatically considered an inconsistency or a reason for exclusion. Instead, variants with incomplete information were reviewed manually.

S3.4 Manual review of flagged variants

All flagged variants were reviewed against the original publication, including its supplementary information, tables, and figures. Reported nucleotide and protein designations were compared with available RefSeq transcripts, the GRCh38 reference sequence, ClinVar, other publications reporting the same variant, and curated variant resources including Franklin by Genoox and VarSome.

Some publications reported only a protein-level designation without a transcript, nucleotide change, or genomic coordinates. In these circumstances, the variant was retained if the reported protein designation could be linked unambiguously to a consistent nucleotide or genomic representation in another publication or curated resource. These variants were flagged as "Cross-checked nomenclature; genomic information unavailable in the original source".

If the reported designation could not be reconciled with the annotation and no independent source provided an unambiguous mapping, the variant was classified as unresolved and excluded from quantitative variant-level analyses. Variants were therefore not excluded solely because genomic information was absent from the original publication. For detailed summary of audited data, see **Supplementary Audit Material.**

The final QC outcomes were mapped back to the carrier-level dataset. All affected variant-class counts, tables, figures, and downstream analyses were regenerated using the audited dataset.

S3.5. Audit code and reproducibility

The R code used to perform the variant-level consistency checks, generate QC flags, map audited variants back to the carrier dataset, and regenerate the summary counts is available at https://github.com/computational-neurogenomics/compound_het_DI_review.git
The repository includes the audit script, a description of the required input fields, a data dictionary for the QC variables, software and database versions, and instructions for reproducing the audit outputs.
